# Evaluation of the surface properties of hair with acoustic emission analysis

**DOI:** 10.1111/ics.12672

**Published:** 2020-11-30

**Authors:** R.L. McMullen, T. Schiess, L. Kulcsar, L. Foltis, T. Gillece

**Affiliations:** ^1^ Ashland Specialty Ingredients, G.P. 1005 US HWY 202/206, Building N Bridgewater NJ USA

**Keywords:** hair, acoustics, surface properties, hair damage, conditioning

## Abstract

**Objective:**

The tactile sensation of hair is an important consumer‐perceivable attribute. There are limited instrumental options to measure the haptic properties of hair. In this study, we introduce a novel technique using the acoustic emissions produced when skin comes in contact with dry hair in a stroking motion.

**Methods:**

Using a free‐field microphone with a frequency response of 8–12,500 Hz, we recorded acoustic emission data of the interaction of skin with hair. Data were captured with Electroacoustics Toolbox software and analysed with Matlab. Acoustic emission profiles were generated allowing us to monitor the acoustic response at distinct frequencies.

**Results:**

Various experiments were conducted to develop this novel technique as a suitable measure to monitor the surface properties of hair. Increasing the normal force and velocity of the interaction led to an increase in acoustic emissions. We also examined the acoustic profile of hair that underwent chemical treatment. For example, bleached hair produced a much higher magnitude acoustic response than the corresponding virgin hair. On the other hand, hair conditioner systems mitigated the acoustic response. Finally, investigations of textured hair revealed that the three‐dimensional structure of the hair fibre assembly and its ability to return to its original state when perturbed produce the most dominant acoustic response for this type of hair.

**Conclusion:**

We introduce a cutting‐edge method to reproducibly evaluate the surface properties of hair. Different types of hair geometry produce unique acoustic profiles as do hair types that experience harsh damaging treatments. This is also a very practical and efficient way to evaluate the degree of protection or conditioning of the fibre.

## Graphical abstract

We developed an acoustic emission technique for measuring the sounds generated when two fingers slide along the length of a hair tress. Using acoustic analysis software, we captured their acoustic profiles and were able to differentiate between virgin and bleached straight hair (European dark brown). In addition, hair with increasing degrees of curvature (e.g., African, mulatto, etc.) produces a greater acoustic response.

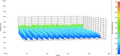

## Résumé

**Objectif:**

La sensation tactile des cheveux est un attribut important perceptible par le consommateur. Il existe des options instrumentales limitées pour mesurer les propriétés haptiques des cheveux. Dans cette étude, nous introduisons une nouvelle technique utilisant les émissions acoustiques produites lorsque la peau entre en contact avec les cheveux secs dans un mouvement de caresses.

**Méthodes:**

En utilisant un microphone champ libre avec une réponse en fréquence de 8 à 12 500 Hz, nous avons enregistré des données d'émission acoustique de l'interaction de la peau avec les cheveux. Les données ont été capturées avec le logiciel Electroacoustics Toolbox et analysées avec Matlab. Nous avons généré des profils d'émission acoustique. De cette manière nous pourrions surveiller la réponse acoustique à des fréquences distinctes.

**Résultats:**

Nous avons fait diverses expériences pour développer cette nouvelle technique comme mesure appropriée pour surveiller les propriétés de surface des cheveux. L'augmentation de la force normale et de la vitesse de l'interaction entre la peau et les cheveux ont causé une augmentation des émissions acoustiques. Nous avons également examiné le profil acoustique des cheveux ayant subi un traitement chimique. Par exemple, les cheveux décolorés ont produit une réponse acoustique de beaucoup plus grande amplitude que les cheveux vierges correspondants. D'autre part, les systèmes d’après shampooing ont atténué la réponse acoustique. Enfin, des investigations sur des cheveux texturés ont révélé que la structure tridimensionnelle de l'ensemble de fibres capillaires et sa capacité à revenir à son état d'origine lorsqu'il est perturbé produisent la réponse acoustique la plus dominante pour ce type de cheveux.

**Conclusion:**

Nous introduisons une méthode de pointe pour évaluer de manière reproductible les propriétés de surface des cheveux. Différents types de géométrie de cheveux produisent des profils acoustiques uniques, tout comme les types de cheveux qui subissent des traitements agressifs. C'est également une manière très pratique et efficace d'évaluer le degré de protection ou de conditionnement des cheveux.

## Introduction

The surface properties of hair are very important from a consumer sensorial perspective and generally reflect the health state of the fibres. The chemistry and morphology of the hair surface play a large role in the haptic perception of hair [1]. Haptics involves the sense of touch and deals with sensorial perception when our skin comes into contact with other objects. The interaction of skin with hair can involve a variety of tactile sensations including roughness and smoothness, which can manifest themselves in the frictional properties of hair.

Hair softness, smoothness and friction are important attributes for the consumer that have challenged scientists in the cosmetic industry to develop representative laboratory techniques able to predict the overall properties of a hair fibre assembly. At the nanoscale, there has been considerable interest in determining the frictional properties of hair fibres. Initial work in this area aimed to establish a relationship between nanotribology of the hair surface and its macroscale properties [2]. Studies conducted in the following years supported and provided a better understanding of the role played by 18‐methyleicosanoic acid in hair structure as well as the overall tribological properties of the fibre [3, 4, 5, 6, 7, 8]. In addition, the interaction of hair fibres with each other as well as with the skin was also investigated [9, 10].

Historically, there was a significant amount of research focused on monitoring the frictional properties of wool and other textile fibres [11, 12, 13, 14, 15, 16]. Much of this interest stemmed from the need to understand the effects of textile processing (e.g., shrink proofing) on the physical properties of the wool fibre [17]. Later on, some of this technology was used to better understand the physicochemical properties of human hair fibres and how this translates into bulk fibre assembly properties, such as hand or feel [18, 19].

Surprisingly, there are relatively few techniques readily available to evaluate the dry surface properties of hair [20, 21, 22]. The most accessible technique is dry combing analysis, which utilizes a load cell to monitor the forces encountered when a comb is dragged through a hair tress [23, 24, 25]. While this technique works extremely well for wet combing analysis, in the dry state it is more difficult to discern differences between treatments and even between virgin and damaged hair. Essentially, dry combing analysis measures frictional forces between the comb and the fibres, frictional forces between fibres, adhesive forces between fibres, torsional and bending moduli resulting from the interaction of the comb with the fibres, and entanglements of the fibres [24, 26]. While this technique is very useful, it is hampered by lack of reproducibility and it is difficult to employ when examining textured hair types.

In this article, we demonstrate a novel technique for probing the surface properties of hair. Acoustics analysis of hair was conducted by recording the acoustic events produced when human skin comes into contact with dry hair by a stroking motion. This technique provides a semi‐quantitative measurement of an otherwise subjective, sensorial property. There have been a number of studies on the analysis of the sound absorption characteristics of fabrics [27, 28, 29]. This mostly stems from interest in utilizing fabrics as acoustic insulators. From a slightly different perspective, the analysis of sounds emitted by fabrics provides information about the sensory perception of fabrics. Along these lines, an acoustic technique was developed to assess the friction and microstructure of model and apparel fabrics [30]. Acoustic emission measurements have also been carried out to provide information about the tactile properties and health state (e.g., ageing) of human skin [31, 32].

Several years ago, a technique for evaluating the surface properties of hair was presented at the 29th IFSCC Congress in Orlando, Florida, USA [33]. Essentially, frictional force was measured with a rub tester and this information was converted into an acoustic melody. In this test, low friction produces lower frequency pitches while high friction results in a higher frequency acoustic signal. The reader should bear in mind that this technique is fundamentally based on friction and the acoustics are produced as a means to convey information about the physical state of the hair surface to the consumer or panelist. In the current study, we focus on acoustic events produced from physically manipulating the hair, which are associated with the surface condition of the fibres.

### Fundamentals of friction

Friction is the force of resistance that an object encounters either in the static state or during motion. For example, if we imagine two surfaces in contact with each other, physical asperities or differences in chemistry at the surface of the two objects would cause resistance to their relative sliding motion. Friction results in the dissipation of energy and increases microcontacts between two surfaces. In cases of extreme stress—such as that found in motors, machine elements, etc.—microfractures and surface degradation (surface wear) can occur. In many engineering applications, friction reduction is a principal objective during the design of materials. However, there are cases which require friction, such as playing a musical instrument (e.g., the violin) or even driving an automobile (friction between the tires and the road keep the automobile stabilized).

Adhesion plays an important role in friction. While little adhesion is normally present between two hard surfaces, introducing a soft surface increases the adhesive forces between the two surfaces. ‘Sticktion’ is a term used to describe a situation where sticking (from adhesive forces) and friction are present. Capillary force is another element affecting friction. It results from a thin layer of fluid on the contact surface. In machined componentry, high humidity can lead to a thin layer of water on the surface leading to capillary forces that could affect the proper mechanical operation of a device.

Historically, Leonardo da Vinci (AD 1452–1519) is often credited with being the first to study in detail and formulate the laws of friction [34]. He established that frictional force is proportional to the normal force, but independent of the contact surface area. These principles were reestablished by the French physicist Guillaume Amontons (AD 1663–1705), which led to what we recognize today as Amontons’ Law:

$$F_{f}=\mu F_{N}$$

where $F_{f}$ is the frictional force, μ is the coefficient of friction and $F_{N}$ is the normal force.

Another important figure in the development of our understanding of dry friction was Leonhard Euler (AD 1707–1783), who established the distinction between static friction and kinetic friction. Static friction is the frictional force between two objects when they are at rest. Essentially, static friction is the force that prevents an object from initially moving when in contact with another. On the other hand, kinetic or dynamic friction describes the frictional forces between two objects in motion. Equation 1 can be written in terms of both static and kinetic friction, where in each case there is a distinct coefficient of friction. Another key contributor to the science of friction was Charles‐Augustin de Coulomb (AD 1736–1806), who determined that dry friction is independent of the sliding velocity of one surface relative to the other—later on, we will see that this is not always the case.

The value of the coefficient of friction depends on the physical and chemical nature of a material. In fact, the coefficient of friction may be considered the summation of the molecular and mechanical component:

$$\mu =\mu_{\mathrm{molecular}}+\mu_{\mathrm{mechanical}}$$

Molecular interactions occur at the outermost surface (surface film), at depths of a fraction of a micron, whereas mechanical interactions take place a little deeper in the substrate surface roughly on the order of several microns [35]. It should also be noted that stick‐slip phenomena are important in friction. As the two surfaces in contact move relative to one another, there is an alternating sliding (slip) and adhesive (stick) behaviour. The sliding of two soft surfaces together often consists of a series of jerky motions.

In this article, we examine the kinetic friction of stroking fingers over the surface of hair in the dry state. Typically, kinetic friction depends on the normal force and velocity at which a frictional pair interact. It also depends on the physicochemical properties of the material under study, such as roughness, hardness and adhesiveness. Friction between two objects can result in thermal emission, optical effects (triboluminescence), low‐frequency vibrations (1 Hz–10 kHz) and acoustic emission (>1 kHz) [35].

### Acoustics of friction

In many engineering applications, noise is caused by friction events. Some of the most common examples in everyday life consist of squealing brakes, squeaking sounds produced by shoes on a floor surface, and chilling sounds resulting from dragging fingernails or chalk on a blackboard. In dynamic friction, one object transfers energy to the other resulting in the dissipation of the energy of motion. When the energy of friction exceeds what the system can dissipate, instabilities in the friction‐excited vibrations or oscillations lead to the production of sound radiation [36].

Therefore, sounds produced by friction are normally transient and not steady. The sounds can emanate from either one or both of the materials in physical contact. In some cases, contact between two substrates can be continuous or transient. In either case, sliding motion is an unsteady process. The interaction forces between two objects determine the types of acoustic pressure waves produced. For example, in the case of weak interactions between the two surfaces, sliding motion should produce an acoustic response that is close to the natural frequency of the substrates. If one or both of the surfaces are rough, the acoustic response may include sounds that are often characterized as roughness noise.

## Materials and methods

Acoustic data were collected with a free‐field microphone (Type 2671 preamplifier and 4188‐A‐021 microphone), manufactured by Brüel & Kjær (Nærum, Denmark), with a frequency response of 8–12,500 Hz. All experiments were carried out in an SE 2000 Series Sound Isolation Enclosure manufactured by Whisper Room, Inc. (Morristown, Tennessee, USA). The microphone was connected to a signal conditioner amplifier (Type 1708, Brüel & Kjær, Nærum, Denmark), which applies gains and filters to the incoming acoustic signal, and then to a USB Audio Interface (ZE 0948, Brüel & Kjær, Nærum, Denmark) connected to MacBook Pro computer (Apple, Cupertino, California, USA). Data were collected with Electroacoustics Toolbox 3.8.5 (Faber Acoustical, LLC, Spanish Fork, Utah, USA) and analysed with Matlab R2017a (Natick, Massachusetts, USA). Data are presented in the form of three‐dimensional acoustic response curves, which provide acoustic magnitude as a function of time and frequency. Calibrations of the microphone and computer system were performed before each experiment with a Type 4231 Sound Calibrator (Brüel & Kjær, Nærum, Denmark) at 94 dB. The noise levels of rooms and locations were measured with an iPhone 6s (Apple, Cupertino, California, USA) using the SPLnFFT Noise Meter application designed by Fabien Lefevre (France) and available for download from the Apple App Store.

Most of the experiments were carried out on European dark brown hair (International Hair Importers & Products, Inc., Glendale, New York, USA) with the dimensions and mass of: L = 18 cm; W = 2 cm; m = 10 g (including a 2.5 cm × 2.0 cm wax tab). In the case of bleached hair, European dark brown hair was subjected to two 1‐h bleaching cycles with 120 g of Clairol Professional BW 2 Powder Lightner (The Wella Corporation, Woodland Hills, California, USA) and 147 mL of Salon Care Professional 20 Volume Clear Developer (Arcadia Beauty Labs LLC, Reno, Nevada, USA). The resulting mixture was applied to damp hair. Experiments were also carried out on African and Brazilian (frizzy) hair purchased from International Hair Importers and Products, Inc. The African hair had the dimensions and mass of: L = 9 cm; W = 10 cm; m = 6.5 g (including a 3.0 cm × 2.75 cm wax tab). The Brazilian hair had the dimensions and mass of: L = 12 cm; W = 14 cm; m = 5.4 g (including a 4.5 cm × 2.0 cm wax tab). All hair was shampooed twice with 3% (w/w) sodium laureth sulphate (SLES):cocamidopropyl betaine (CAPB) (12:2) prior to conducting experiments. SLES (Steol CS‐130) and CAPB (Amphosol CA) were obtained from Stepan (Northfield, Illinois, USA).

**Figure 1:**
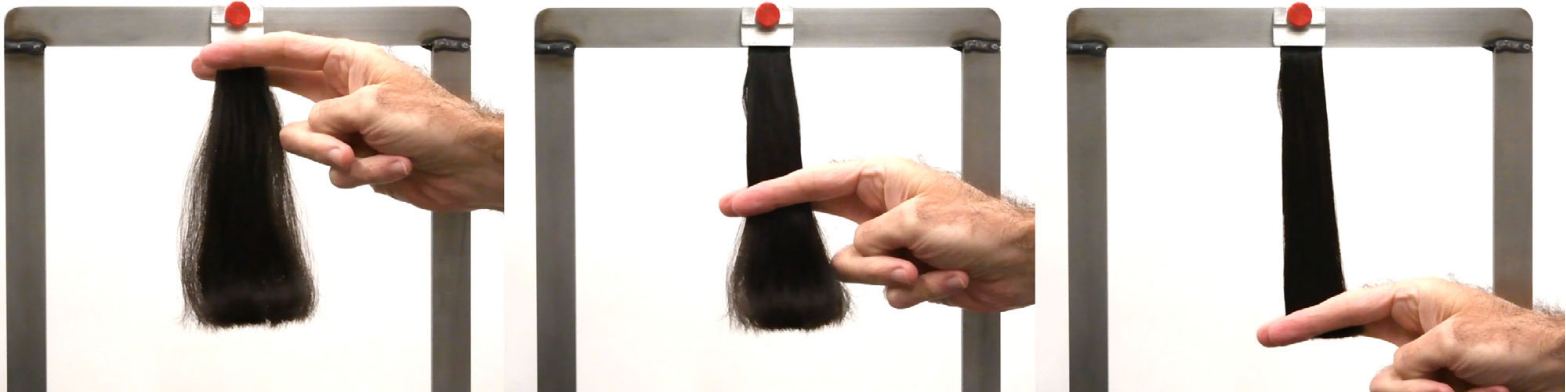
Photographs demonstrating the motion of the index and middle fingers as they pass over the front and rear of the hair tress from top to bottom.

To prepare European dark brown hair for acoustics analysis, a wet hair tress was combed five times with the large teeth of a comb and 5 times with the small teeth to remove tangles. [The comb, Sally Beauty Supply 487305, was purchased from Sally Beauty Supply (Denton, Texas, USA). The comb was characterized by the packing density of the large (31 teeth/8.7 cm) and small (59 teeth/8.7 cm) teeth.]. The tress was then re‐wet with water and the index and middle finger were run along the hair tress with the tress in between the two fingers removing excess water. The tress was then allowed to hang dry for 15 h. In the case of textured hair, the fibres were not detangled as this would distort the natural curl present in the hair fibre assembly. Tresses were allowed to dry in an environment of 40% RH and 25°C. Acoustic measurements were also conducted at these climate conditions. To conduct acoustic emission analysis, hair tresses were mounted on a stainless‐steel frame (see Fig. 1). The microphone was mounted separately and positioned 3 cm down from the top of the tress (below the bottom of the hair tress wax tab) at a distance of 4 cm from the back of the tress.

A Texture Analyzer (TA.XT Plus, Texture Technologies Corp., South Hamilton, Massachusetts, USA) was used to determine the stroking force of the middle and index fingers over the hair tress. The hair tress was mounted to the arm of the Texture Analyzer, and the following settings were employed while maintaining the hair tress between the index and middle finger during testing: test type—1 return to start; test mode—tension; test speed—40 mm/s; target mode—distance; distance—100 mm; trigger type—button. Data were analysed with Exponent software version 6.1.16.0 by Stable Microsystems (Texture Technologies Corp., South Hamilton, Massachusetts, USA).

Prior to measurements, the panelist washed his/her hands with 0.2 g of Palmolive Ultra Strength dish soap (Colgate‐Palmolive, New York, New York, USA) followed by extensive rinsing (1 min) to remove any oils or debris that could interfere with the measurements. They dried their hands thoroughly with VWR Light‐duty Tissue Wipers (VWR, Radnor, Pennsylvania, USA) and then sat in the environmentally controlled room (40% RH and 25°C) for 15 min prior to conducting experiments to ensure that drying reached equilibrium. Fingers were subjected to the washing procedure prior to each treatment group, which consisted of five tresses each for virgin, bleached and conditioner treated hair. Separate experiments were also carried out (data not shown) to ensure that the hand washing procedure did not affect the results by washing the hands before the acoustic measurements of each hair tress for a series of five virgin European dark brown hair tresses.

To evaluate the effects of conditioning treatments on the surface properties of hair, two types of conditioners were studied; one based on a conventional cationic surfactant system containing stearamidopropyl dimethylamine and cetrimonium chloride (Conditioner A), and another containing the cationic surfactants and a cationic polymer (polyquaternium‐113) (Conditioner B). Table 1 provides a list of the ingredients for each conditioner. Hair tresses were treated with 0.5 g of the conditioner prototypes. The conditioner was massaged into the hair for a period of 10 s with the fingers and combed through several times to ensure proper distribution. Hair was then rinsed for 30 s and allowed to air‐dry overnight prior to conducting acoustic measurements. The pH of the formulation prototypes was 3.7.

**Table 1:** Ingredients in Conditioners A and B

Ingredient	Conditioner A	Conditioner B
DI H_2_O	88.21%	87.21
Cetyl alcohol (Cetyl Alcohol NF*)	5.00%	5.00%
Stearyl alcohol (Stearyl Alcohol NF*)	2.00%	2.00%
Cetrimonium chloride (Jeequat CT‐29*)	1.00%	1.00%
Stearamidopropyl dimethylamine (Jeechem S‐13*)	1.00%	1.00%
Dimethicone (Jeesilc 110*)	1.00%	1.00%
Phenoxyethanol (and) caprylyl alcohol (Optiphen Preservative^†^)	0.75%	0.75%
Lactic acid^†^	0.54%	0.54%
Propylene glycol (Propylene Glycol USP*)	0.50%	0.50%
Polyquaternium‐113 (ChromoHance 113^†^)	—	1.00%

Quantities are listed in % (w/w).

Products provided by Jeen International, Fairfield, New Jersey, USA.

Products obtained from Ashland Specialty Ingredients G.P., Wilmington, Delaware, USA.

Product purchased from VWR Scientific, Radnor Pennsylvania, USA.

For the majority of the data presented in this text, one expert panelist carried out the evaluation of the hair tresses. To gain a better understanding of the influence of the panelist on the results, we conducted a pilot study with five panelists in which they compared untreated bleached hair with bleached hair treated with a commercial hair conditioner. The panelists consisted of both males and females from diverse racial backgrounds. Bleached hair was prepared by the method already mentioned and conditioner treatment was carried out by treating each tress with 0.5 g of the commercial conditioner TRESemmé Color Revitalize (Unilever, Trumbull, Connecticut, USA) based on stearamidopropyl dimethylamine and behentrimonium chloride. The same treatment protocol of hair was followed as already discussed for the conditioner formulation prototypes. The pH of the commercial conditioner was 4.3.

## Results and discussion

Mechanical manipulation of hair produces sensorial effects that can be monitored by recording the acoustic emission profile. In the experiments described in this article we demonstrate the use of acoustics to measure the dry sensorial properties of hair when the middle and index finger slide along the length of a hair tress. The acoustic emission properties of virgin dark brown hair were compared to bleached hair. Our results, presented as a function of the various frequencies of acoustic events, demonstrate that chemically damaged hair produces higher magnitude acoustic events. We also compared different types of textured hair—African vs. Brazilian (frizzy) vs. straight hair—and found that the greater the textural properties the more pronounced the acoustic response. In addition, we investigated the effect of several conditioning treatments and found that these can mitigate the surface‐acoustical properties hair.

### Implementation of the acoustic emission technique

Figure 1 contains a photograph demonstrating the technique of sliding the index and middle finger along a hair tress. The hair tress is mounted on a custom‐designed stainless‐steel frame. During the procedure, hair is subjected to forces from the downward motion and the pressure exerted by the fingers on the tress. We discuss the influence of velocity and pressure as the fingers pass over the hair tress further along in this article.

**Figure 2:**
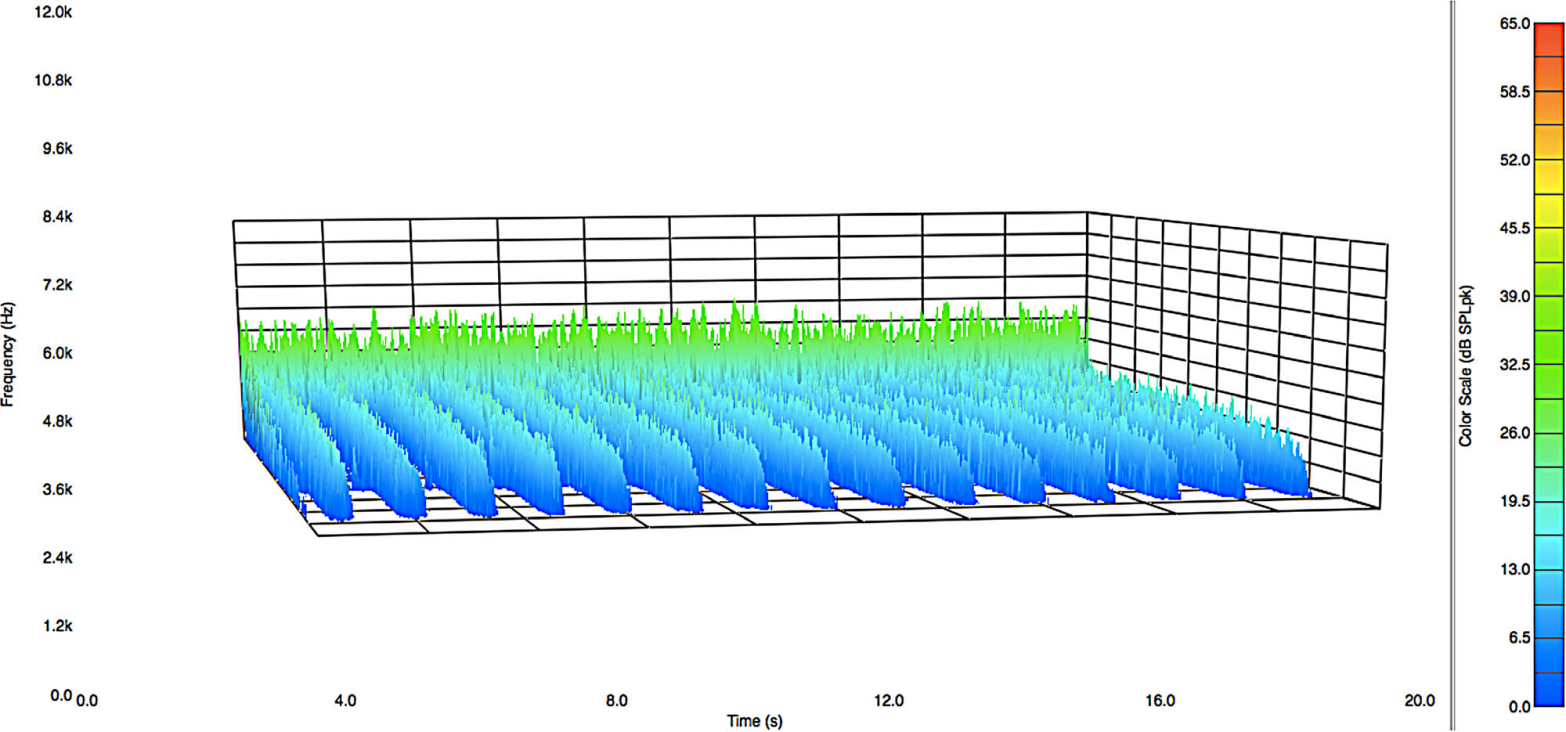
Acoustic emission frequency decomposition plot for European dark brown hair. The average normal force was 39.10 g.

Figure 2 presents data in the form of a frequency distribution plot of the acoustic emission magnitude as a function of time for about 15 strokes along a hair tress. As a result, there are 15 peaks in the acoustic response curve. Low frequency (1000 Hz–4000 Hz) is located in the rear of the plot and high frequency (8000 Hz–12,000 Hz) towards the front.

The acoustic emission magnitude is reported as sound pressure level (SPL) peak (pk) in units of decibels (dB). Table 2 contains a list of sounds and the corresponding decibel level, which were measured at various locations. It should be noted that the magnitude of an acoustic event depends on the distance of the audio recording device from the source of the sound. Essentially, when a sound is created, pressure waves emanate from the source in the form of rarefactions (less dense air molecules) and compressions (more dense air molecules) and are detected by the anatomical ear unit (ear drum, malleus, cochlea, etc.) or a microphone. In each case, a pressure wave interacts with a diaphragm and is converted to an electrical signal.

**Table 2:** Acoustic events and their corresponding magnitude

Acoustic event	Magnitude (dB SPL)
Rock music concert (Boston; ‘Foreplay/Long Time’; Coney Island, NY, USA)	90–118
NYC subway train (Bronx, NY, USA)	80–101
Laboratory with working hoods (Bridgewater, NJ, USA)	53–66
Mt. Olive Public Library (Mt. Olive, NJ, USA)	32–36
Quiet basement (Mt. Olive, NJ, USA)	25–30

Measured with the SPLnFFT Noise Meter application on an iPhone 6.

As already noted, the acoustic response plot in Fig. 2 contains a range of frequencies. The low‐frequency data (*ca*. 8 Hz to 2000 Hz) correspond to background noise and vibrations caused by temperature control units, vacuum hood systems, plumbing, electrical equipment and electronic devices found in a typical research and development building. This low‐frequency noise is nearly impossible to eliminate even when an isolated acoustic chamber is placed in a silent laboratory in the basement of a building. The upper limit of frequency reported depends on the range of the microphone. The microphone used in this study has an upper frequency limit of 12,000 Hz. In the acoustic profile in Fig. 2, there is a decreasing slope from 10,000 – 12,000 Hz. Accordingly, we do not use any data beyond 10,000 Hz in our analyses. The human ear is capable of detecting sounds between 20–20,000 Hz. Typically, once middle age is reached, the ability to hear sounds above 12,000 Hz diminishes greatly. We recently conducted experiments with a higher dynamic range microphone (2.6 Hz–18,000 Hz) that more accurately represents the range of a young and healthy human auditory device and found that the data were equivalent to those with the lower dynamic range microphone (8 Hz–12,500 Hz).

**Figure 3:**
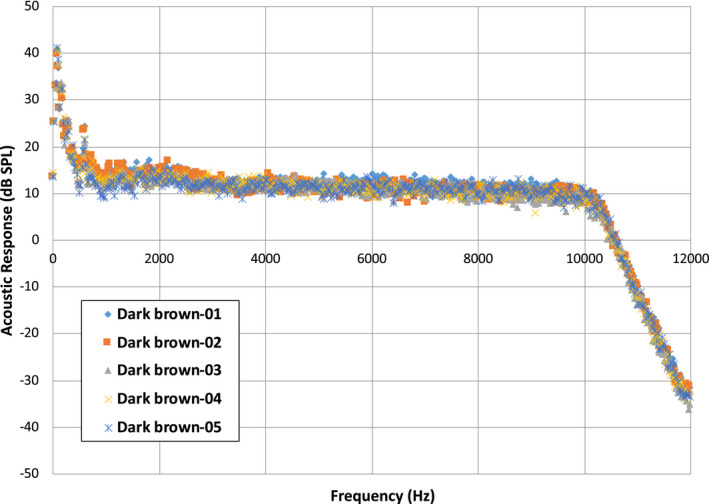
Analysis of frequency distribution for 5 different European dark brown hair tresses. Each point represents the average value of the peak maxima at each frequency from the acoustic emission profile plot.

The data from Fig. 2 were processed with MatLab software to determine the peak maxima at each frequency and provide an average value. Figure 3 contains the output of this computation where the average peak maxima values of acoustic emission are plotted as a function of frequency. Data were collected for five different hair tresses in order to demonstrate the reproducibility of this method. Clearly, a high‐intensity peak can be found at low frequency for all of the tresses. This corresponds to environmental noise and is disregarded in the final analysis. Again, the negative slope beginning at 10,000 Hz and ending at 12,000 Hz is due to the higher frequency limit of the microphone. Therefore, to obtain an overall value for the acoustic response, we typically integrate plots such as those in Fig. 2 from 4000 to 8000 Hz. In the case of the five dark brown hair tresses, we obtained an average value of 48,340 ± 1,623 dB SPL·Hz, which demonstrates very good reproducibility. The corresponding coefficient of variation (CV) is 3.36%.

**Figure 4:**
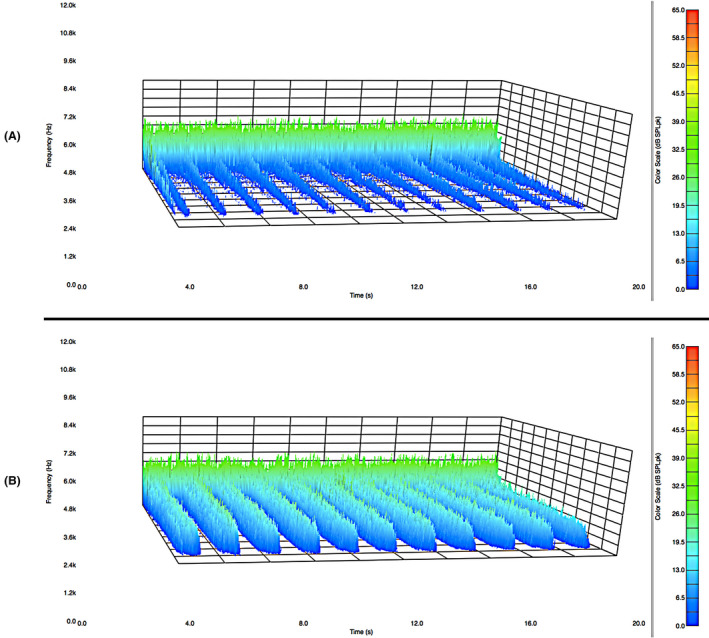
Effect of applied normal force on the acoustic response for (A) low and (B) high applied forces to a European dark brown hair tress corresponding to (A) 11.52 g ± 4.14 and (B) 145.38 g ± 30.78.

Several experiments were conducted to investigate the effect of normal force on the acoustical response. The drag force was measured by attaching a hair tress to a Texture Analyzer containing a load cell, placing the index and middle fingers on each side of the tress, and programming the instrument to pull the tress a distance 100 mm while monitoring the force. As already stated, three different forces—low (11.52 g ± 4.14), medium (39.10 g ± 12.20), and high (145.38 g ± 30.78)—were applied by the fingers on the tress. In general, we found that the applied forces could be reproduced by trained panelists to within 8%–35% of the average applied force for each defined pressure. At lower applied forces, there is less reproducibility of the application force by the fingers on the hair tress. Figure 4 contains acoustic emission plots for (a) low and (b) high applied forces. It is clearly demonstrated in the plot that there is a large difference in the magnitude of the acoustic emission between the two applied forces.

**Figure 5:**
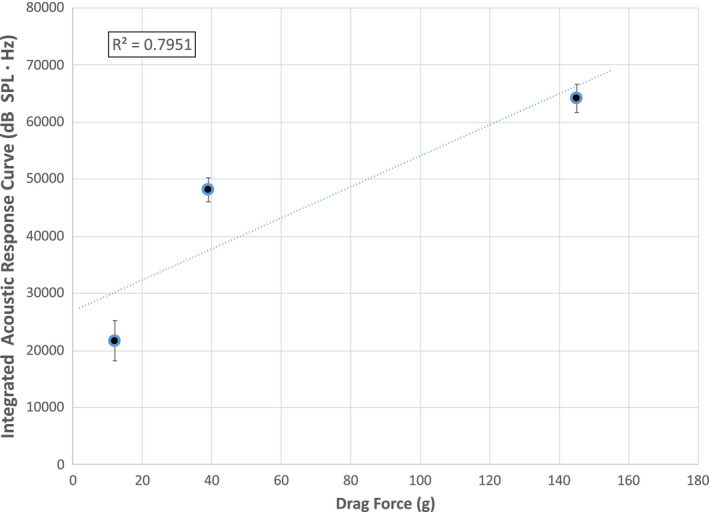
Integrated acoustic response (4000–8000 Hz) for three applied normal forces. Experiments were conducted on European dark brown hair tresses.

Figure 5 contains a plot of the integrated values from the acoustic response curves (i.e. integration of curves such as those shown in Fig. 3 from 4000 to 8000 Hz) for the three applied forces examined. As expected, increasing the applied force results in higher integrated acoustic response curve values. The linearity of the relationship is rather low (*R* ^2^ = 0.795); however, the standard deviation for each individual measurement is also low. Since only three points were obtained for this data set, a fourth or fifth point might result in a higher *R* ^2^ value, or demonstrate a non‐linear response. Each value on the curve represents the average obtained for two European dark brown hair tresses. Overall, this suggests deviation from Amontons’ law. However, it should be pointed out that this assumption implies that sound and friction are linearly related (i.e. sound measures the force of friction), which is debatable, especially for complex systems. In comparison, it has been shown that woven and non‐woven textiles deviate from Amontons’ law and a power law can be used to more accurately describe the relationship between applied force and coefficient of friction [37, 38]. In the case of wool, it was reported that Amontons’ law is somewhat relevant at low applied loads [39]. In addition to the chemistry and microstructure, the frictional behaviour of hair and wool is highly influenced by the scale‐like nature of the outermost cuticle layer, which certainly affects the macroscale friction.

**Figure 6:**
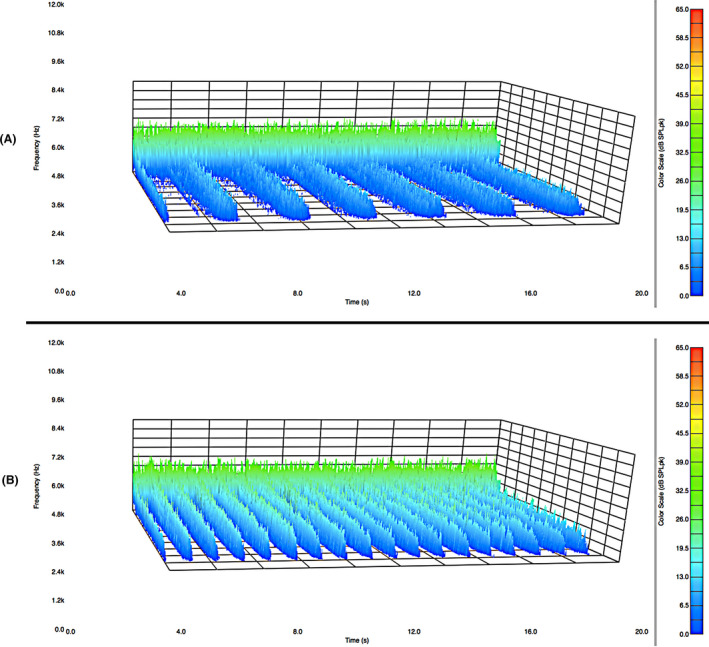
Effect of velocity on the acoustic response for stroke rates along the hair tress of (A) 0.35 strokes/s and (B) 0.88 strokes/s. Experiments were conducted on European dark brown hair tresses. The average normal force was 39.10 g.

The effect of velocity along the hair tress was also examined. Stroke rates of 0.35, 0.63 and 0.88 strokes/s along the hair tress revealed an increase in the acoustic response when the rubbing velocity increases. The acoustic emission plots for (a) 0.35 and (b) 0.88 strokes/s are provided in Fig. 6. A large increase in acoustic magnitude (across all frequencies) at higher rubbing velocity is apparent in the figure. At the lower stroke velocity, the acoustic peaks are broader and have a lower magnitude.

**Figure 7:**
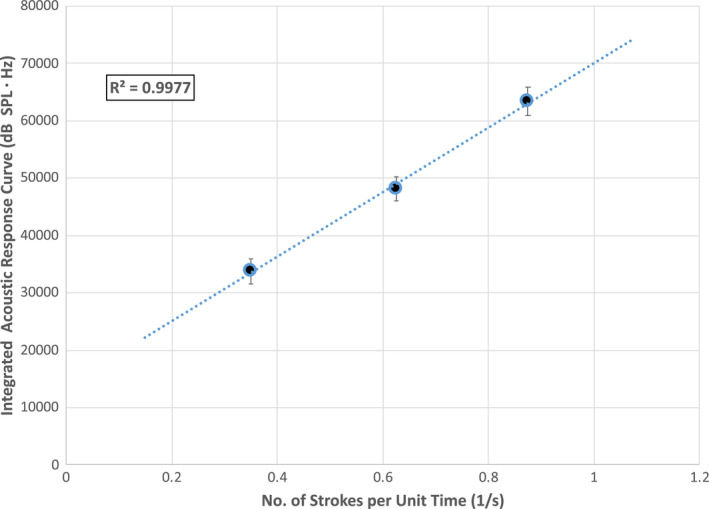
Integrated acoustic response (4000–8000 Hz) for three stroke rates: 0.35, 0.63, and 0.88 strokes/s. Experiments were conducted on European dark brown hair tresses. The average normal force was 39.10 g.

To better illustrate and summarize the effects of stroke velocity on acoustic emission magnitude, Fig. 7 contains a plot of the integrated values from the acoustic response curves (i.e. integration of curves such as those shown in Fig. 3 from 4000 to 8000 Hz) for three different rubbing velocities. For this data set, each value represents the average obtained for two European dark brown hair tresses. The linearity of the relationship between the integrated acoustic response and stroke velocity is impressive (*R* ^2^ = 0.998) as well as the extremely low standard deviation. These data disagree with Coulomb’s law, which states that dry friction should be independent of the sliding velocity. This assumption is often assumed for simple cases and applies to elastic materials, such as metals. Viscoelastic materials, such as hair and fabrics, often demonstrate friction dependence on velocity, deviating from Coulomb’s law. In this particular case, the interaction is between skin and hair, which introduces another level of complexity, and could lead to the adaptation of the skin texture to the hair. Again, similar to the case with Amontons’ law, we should point out that we make the assumption that the acoustic and friction response are linearly related.

### Effect of bleaching on the acoustic emission profile of hair

In addition to changing the pigmentary profile of hair, bleaching has a number of side effects that result in damage to its morphological structures. For example, it is well known that bleaching treatments oxidize disulphide residues resulting in the formation of cysteic acid and also damage other protein structures in hair [40]. In addition, the fibre becomes extremely porous and contains cracks, crevices and other asperities throughout its structure [41]. The surface of hair is also extremely sensitive to bleaching. It has been reported that covalently attached 18‐methyleicosanoic acid—a lipid that in large part is thought to be responsible for conferring the fibre with a more lubricious surface—might be damaged or removed as a result of bleaching [4]. Dynamic contact angle measurements have clearly demonstrated the transition of the hair surface from a hydrophobic to hydrophilic environment as a result of the bleaching process [42]. Overall, bleached hair has a very raspy feel compared to virgin hair.

**Figure 8:**
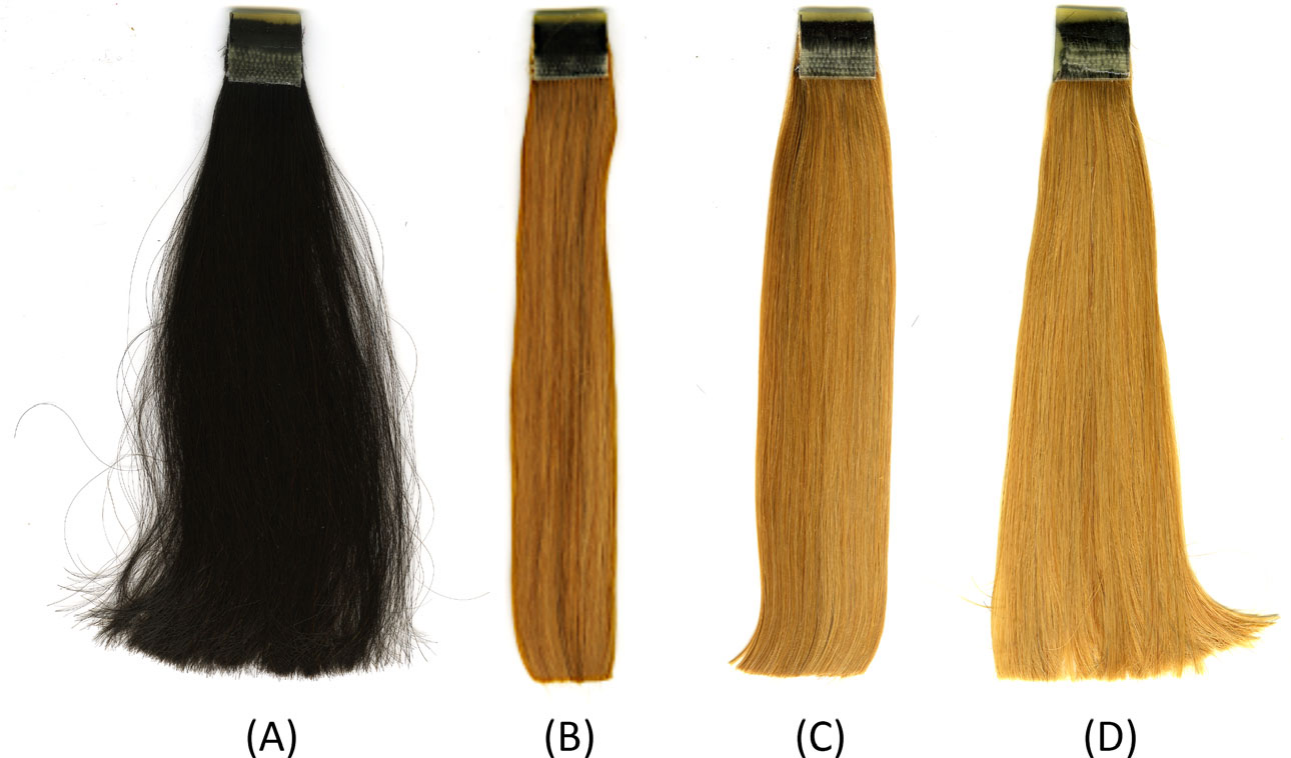
Photograph of (A) European dark brown virgin hair, (B) bleached hair, (C) bleached hair treated with Conditioner A and (D) bleached hair treated with Conditioner B.

In this study, we compared the acoustic emission of European virgin dark brown hair with the same lot of hair that underwent a bleaching procedure and then was treated with two different conditioner prototypes. Figure 8 contains a photograph portraying European dark brown virgin hair, bleached hair, bleached hair treated with Conditioner A and bleached hair treated with Conditioner B after they were allowed to dry overnight (15 h) at 50% RH and 25°C. As seen in Fig. 8, bleaching hair introduces inter‐fibre binding rendering the tress more rigid, which was confirmed by cantilever bending experiments. Treatment of hair with conventional (Conditioner A) or polymeric conditioners (Conditioner B) results in separation (less binding) of the fibres and gives the hair a softer, fluffy feel as described by panelists.

**Figure 9:**
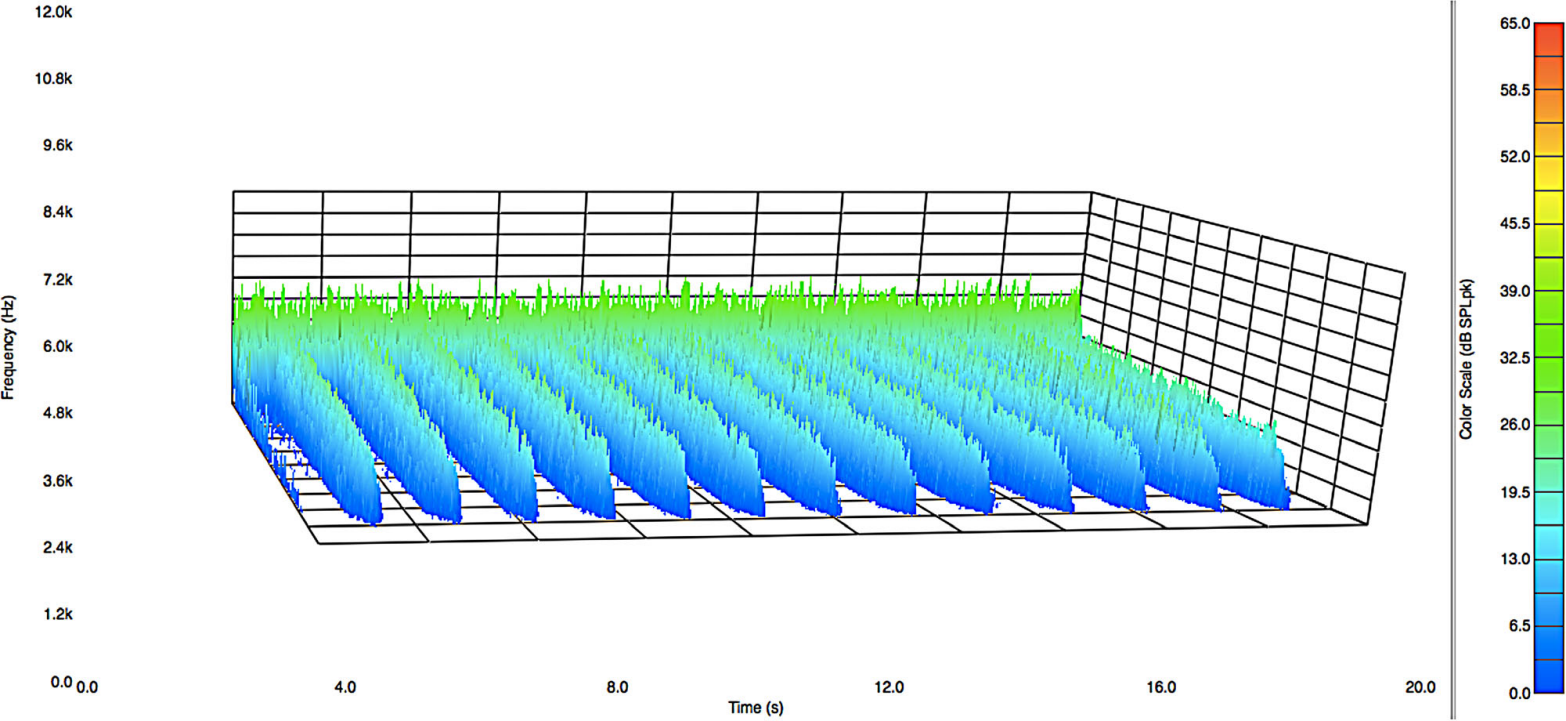
Acoustic emission frequency decomposition plot for bleached hair. The average normal force was 39.10 g.

Figure 9 contains an acoustic response plot of bleached hair. Immediately apparent in the graph are the higher intensity peaks as compared to virgin dark brown hair presented in Fig. 2. In the current study, the increase in acoustic emission from bleached hair suggests that structurally induced changes result in a higher friction surface. More than likely, changes in the acoustic response could result from modifications in the chemistry of the surface (removal of bound and/or non‐bound lipids, oxidation of proteins, etc.) and changes in the microstructure of the cuticle surface.

**Figure 10:**
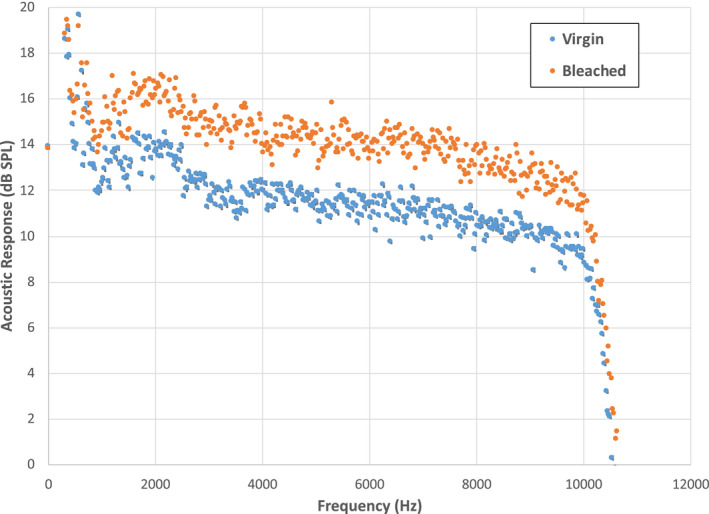
Analysis of frequency distribution for (A) virgin European dark brown and (B) bleached hair. The average normal force was 39.10 g.

To more clearly discern the differences between virgin and bleached hair, Fig. 10 contains a plot of the respective frequency distributions. In the same manner as Fig. 3, these plots are constructed by obtaining an average value of the peak maxima values at a particular frequency. The acoustic emission at all frequencies is clearly higher for bleached hair as compared to virgin hair. Integration of acoustic response curves (such as that shown in Fig. 10) for virgin and bleached hair from 4000 to 8000 Hz, yields values of 51,378 ± 2642 dB SPL·Hz (CV = 5.14%) and 67,912 ± 3493 dB SPL·Hz (CV = 5.14%), respectively, which is a 32% increase in acoustic magnitude. Note that the coefficient of variation is the same for both sets of data.

The same analysis carried out for Conditioner A and Conditioner B resulted in 52,945 ± 1952 dB SPL·Hz (CV = 3.69%) and 53,636 ± 2662 dB SPL·Hz (CV = 4.96%), respectively. Hence, Conditioner A and B perform similarly, although we would expect slightly higher values for Conditioner B since it contains a polymeric material, which generally does not provide hair with the same deep conditioning as a cationic surfactant. Please note that the values reported for virgin, bleached, Conditioner A and Conditioner B were obtained by averaging the integrated acoustic response curves obtained for five different hair tresses. The CV is lower for both of these samples than for virgin and bleached hair, which might reflect more uniformity in the surface properties as a result of the treatments. The polymeric conditioner contains a slightly higher CV value than cationic surfactant conditioner, which may result from a greater number of stick‐slip events and/or less uniform coverage of the surface. Typically, cationic polymers are added as supplemental conditioning agents to conditioners since they provide a more durable, longer lasting conditioning effect, which is resistant to shampooing.

**Figure 11:**
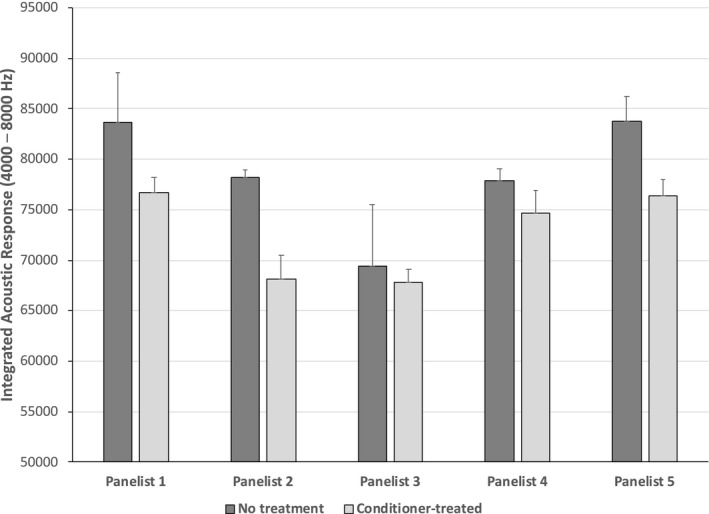
Integrated acoustic response (4000–8000 Hz) for untreated, bleached European dark brown hair and bleached European dark brown hair treated with a commercial conditioner.

To investigate the effect of the panelist on the acoustic evaluation, we measured the acoustic response produced by untreated bleached hair and bleached hair treated with a commercial conditioner based on stearamidopropyl dimethylamine and behentrimonium chloride. The results for this analysis (Fig. 11) demonstrate that there are differences in the magnitude of the acoustic response depending on the panelist. However, the same trend was observed by nearly all of the panelists where untreated bleached hair produced a greater acoustic response than the same hair type treated with a commercial conditioner.

### Acoustics analysis of textured hair

**Figure 12:**
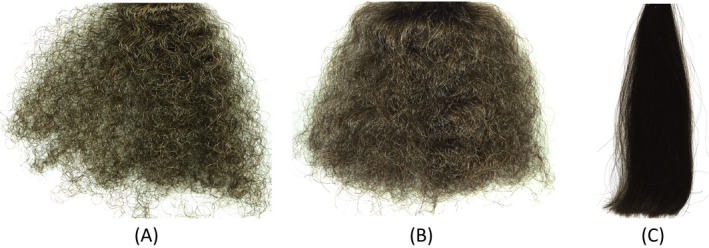
Photograph of (A) tightly curled African, (B) frizzy Brazilian and (C) straight European dark brown hair.

A large majority of the population has textured hair. Textured hair refers to hair characterized by a certain degree of curliness, frizziness, waviness or kinkiness. To better understand the effects of the degree of texture on the acoustic response for this method, we investigated African (tightly curled) and Brazilian (frizzy) hair, comparing them to straight European dark brown hair. Figure 12 contains a photograph of the three hair types where one can gain a perspective of the three‐dimensional fibre assembly.

**Figure 13:**
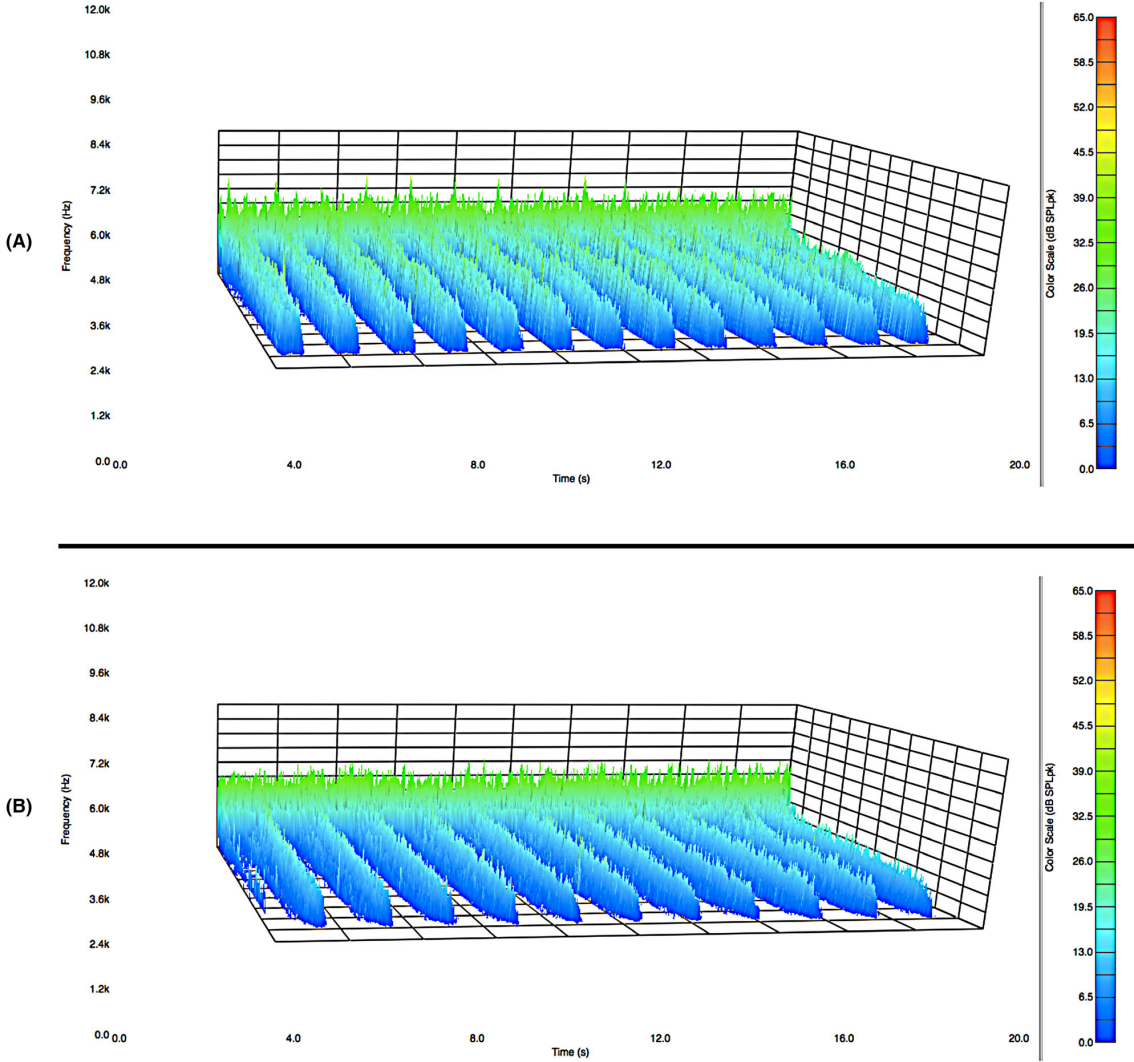
Effect of hair texture on the acoustic response: (A) tightly curled African and (B) straight European dark brown hair. The average normal force was 39.10 g.

The corresponding acoustic response plots are provided in Fig. 13. The magnitude of the acoustic response of the tightly curled African hair is significantly higher than that for straight hair. If the plots in Fig. 12 are processed to determine the peak height average at all of the different frequencies, we can integrate the acoustic response between 4000 and 8000 Hz as described above. As a result, we find the following values for the three different types of hair texture: tightly curled African (73,021 dB SPL·Hz ± 1250; CV = 1.71%), frizzy Brazilian (58,949 dB SPL·Hz ± 1420; CV = 2.41%) and straight European dark brown hair (52,790 dB SPL·Hz ± 2810; CV = 5.32%). Please note that values are obtained by averaging integrated acoustic response curves obtained for one hair tress measured two times (African and frizzy hair). In the case of straight European dark brown hair, average values represent data obtained for five different tresses measured once. The CV values for African and Brazilian hair are lower than European dark brown hair. One explanation may be that only one tress was used to measure the acoustic emissions of African and Brazilian hair, whereas five tresses represent the average reported for European dark brown hair. The consistency of the applied force on the tress probably also plays a major role in the evaluation of CV.

**Figure 14:**
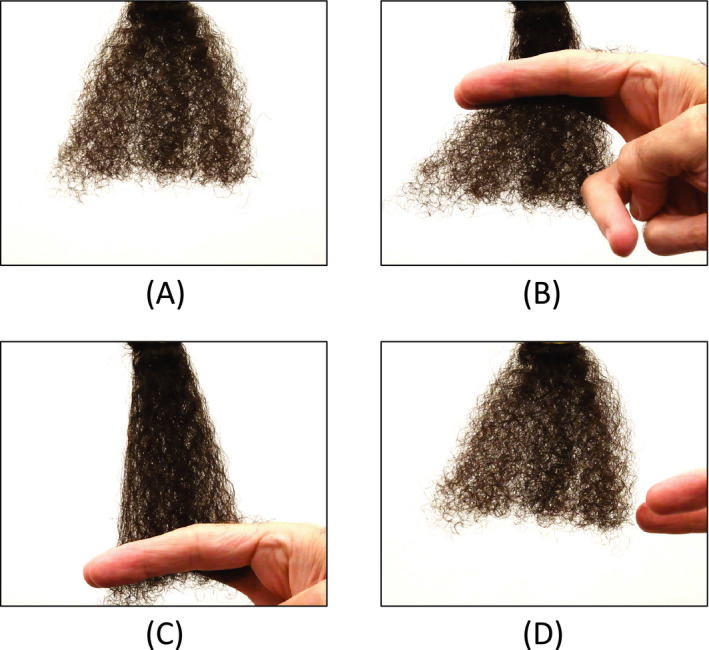
Stroking of textured hair with the index and middle fingers: (A) undisturbed tress, (B) tress at the beginning of the stroke, (C) tress at the end of the stroke and (D) tress after the stroke. Note the change of shape of the tress during the stroke and its return to its original position after stroking.

More than likely, the increase in the magnitude of the acoustic response for higher degrees of hair texture is due to the geometrical three‐dimensional arrangement of the fibre assemblies. In addition, as shown in Fig. 14, there is a notable change in the shape of textured hair during the stroking process. This change is temporary and the hair reverts back to its original shape after stroking. This snap‐back behaviour produces a pressure wave, resulting in a sound and acoustical signal. Therefore, when analysing textured hair, the acoustic emission results from both the surface chemistry characteristics of the fibres as well as the three‐dimensional arrangement of the fibre assembly and its ability to return to its original position (elasticity).

## Conclusions

A novel acoustic technique is presented that allows for the semi‐quantitative evaluation of the sensorial properties of human hair, which can be likened to stroking or caressing the hair. We compared damaged versus undamaged hair and found significant differences in their acoustic emissions. Analysis of hair treated with a conventional and polymeric conditioning system illustrated that treatments are able to ameliorate frictional affects introduced by bleaching hair. In addition, we observed notable distinctions in different textural hair types. Unlike straight hair, a large contributing factor to the acoustic emissions produced by textured hair is its ability to change its three‐dimensional orientation and return back to its original shape. The acoustic profile of straight hair, on the other hand, depends more on the surface properties of the fibre.
